# Potential Role of Pet Cats As a Sentinel Species for Human Exposure to Flame Retardants

**DOI:** 10.3389/fvets.2017.00079

**Published:** 2017-05-31

**Authors:** Luis A. Henríquez-Hernández, Elena Carretón, María Camacho, José Alberto Montoya-Alonso, Luis D. Boada, Verónica Bernal Martín, Yaiza Falcón Cordón, Soraya Falcón Cordón, Manuel Zumbado, Octavio P. Luzardo

**Affiliations:** ^1^Toxicology Unit, Research Institute of Biomedical and Health Sciences (IUIBS), Universidad de Las Palmas de Gran Canaria, Las Palmas, Spain; ^2^Internal Medicine Service, Faculty of Veterinary Medicine, Research Institute of Biomedical and Health Sciences (IUIBS), Universidad de Las Palmas de Gran Canaria, Las Palmas, Spain; ^3^Spanish Biomedical Research Centre in Physiopathology of Obesity and Nutrition (CIBERObn), Las Palmas, Spain

**Keywords:** persistent organic pollutants, polychlorinated biphenyls, organophosphorus compounds, brominated flame retardants, pets, biomonitoring of pollutants

## Abstract

Flame retardants are a wide group of chemicals used by the industry to avoid combustion of materials. These substances are commonly found in plastics, electronic equipment, fabrics, and in many other everyday articles. Subsequently, ubiquitous environmental contamination by these common chemical is frequently reported. In the present study, we have evaluated the level of exposure to polychlorinated biphenyls (PCBs), brominated diphenyl ethers (BDEs), and organophosphorous flame retardants (OPFRs) in pet cats through the analysis of their serum. We also analyzed the level exposure to such chemicals in a series of 20 cat owners, trying to disclose the role of pet cats as sentinel species of human exposure to FRs. Our results showed that PCBs, banned 40 years ago, showed the lowest levels of exposure, followed by BDEs—banned recently. Congeners PCB-138 and PCB-180 were detected in ≥50% of the series, while BDE-47 was detected in near 90% of the pet cats. On the other hand, the highest levels were that of OPFRs, whose pattern of detection was similar to that observed in humans, thus suggesting a potential role of cats as a sentinel species for human exposure to these currently used FRs. Six out of 11 OPFRs determined [2-ethylhexyldiphenyl phosphate, tributylphosphate, triisobutylphosphate, triphenylphosphate, tris (2-chloroethyl) phosphate, and tris (2-chloroisopropyl) phosphate] were detected in 100% of the samples. It will be interesting to perform future studied aimed to elucidating the potential toxicological effects of these highly detected chemicals both, in cats and humans.

## Introduction

Flame retardants (FRs) are chemical additives in materials to prevent combustion and to delay the spread of fire after ignition (1). There are two types of FRs, the additive FRs, which are mixed with the base material and are mainly of mineral origin, and reactive FRs, which are bonded to the base material, and are usually organohalogenated or organophosphorus compounds (2). Many halogenated chemicals, such as some brominated flame retardants (BFRs) and polychlorinated biphenyls (PCBs), are environmentally persistent, bioaccumulative, and/or toxic in the environment, as well as toxic to animals and humans. Phosphate flame retardants (PFRs) have been used commercially since the 1970s in a variety of products as possible substitutes for bromine-containing formulations (3), and currently these are the most used worldwide.

Polychlorinated biphenyls are polyhalogenated aromatic hydrocarbons that have been widely used in a variety of applications: closed systems such as electrical capacitors and transformers, paints, rubbers, glues, wire coatings, insulating tapes, thermal paper, fungicides and other pesticides, or fertilizers among many others (4). These compounds are volatile and evaporate easily from water surfaces and their movement through the atmosphere, resulting in widespread dispersal into the environment (5). Moreover, due to their chemical characteristics they accumulate (and biomagnify) in the food chain and the ingestion of PCBs with food in animals and human being has been reported to be relevant, leading to bioaccumulation throughout their lives (6). PCB production decreased and eventually ceased in the 1970s (7). In most Western countries (including Spain), PCBs were banned in the late 1980s (8, 9). However, thousands of tons are still releasing from obsolete electric and electronic equipment (10, 11), and detectable levels of such compounds are nowadays found in serum of humans (12) and other animals (13).

Brominated flame retardants (BFRs) are industrial chemicals produced to fire-protect construction materials, indoor decorations, furniture, textiles, electronics, and electrical appliances (14). One of the major classes of BFRs are the polybrominated diphenyl ethers (poly-BDEs) (15). Unlike PCBs, for which it has been established that dietary intake is the most important route of exposure, BFRs also have indoor dust as a significant source of exposure via inhalation and hand-to-mouth activities, of special concern for small children (16) and cats (17). Despite the fact that the Stockholm Convention on Persistent Organic Pollutants listed some BDEs (tetra-, penta-, hexa-, and hepta-BDEs) in Annex A—elimination—they are often detected in the environment (13). As PCBs, BDEs are considered endocrine disrupters (18) and have been associated with the development of diseases in humans and also in other species such as pet cats (19).

Phosphate flame retardants can be divided in three main groups: inorganic PFRs, organic PFRs (including organophosphate esters, phosphonates, and phosphinates), and halogenated PFRs (20). Organophosphorous flame retardants (OPFRs) are currently considered as more environmentally friendly and safer than the brominated-based flame retardants. As a consequence, PFRs are being increasingly employed in consumer products. However, chemicals such as tris(2-chloroethyl)phosphate (TCEP) or tris(1,3-dichloro-2-propyl)phosphate (TDCPP) are considered dangerous for the environment (21–23). Although PFRs have been present in industrial formulations since 40 years ago, information about their environmental fate or their effects in humans and biota are still scarce.

The aim of the present study was to assess the serum levels of PCBs, BDEs, and OPFRs in pet cats from Gran Canaria (Canary Islands, Spain). Since cat pets have been closely associated with the indoor environment, and besides due to their grooming habits, cats have been classically used to evaluate the level of human exposure to BDEs through the indoor dust, serum levels of such compounds were also determined in a series of humans that share environment with them, trying to evaluate the correlation among species, and to disclose whether pet cats may be sentinels of human contamination by these pollutants.

## Materials and Methods

### Sampling

Cat blood samples were prospectively collected between October and November 2016. The blood was extracted through cephalic vein puncture. Twenty-two pet cats visiting the Veterinary Hospital of the Faculty of Veterinary of the University of Las Palmas de Gran Canaria (Canary Islands, Spain) for routine care were included in this study. We employed heparinized tubes for the blood extraction, and serum was immediately obtained by centrifugation. Serum samples were kept frozen at −20°C until sample preparation for chemical analysis.

Human blood samples (*n* = 20) were collected from volunteers, which fulfilled the condition of being cat owners (although not the owners of these cats), and being residents in Gran Canaria. Blood collection of these volunteers took place between October and December 2016 in the Insular Hospital (Gran Canaria, Canary Islands, Spain). All human volunteers provided their written informed consent to participate in this study. The cat owners also signed an informed consent of the participation of their pet cats in this study. We obtained the approval from the Research and Ethics Committee of our Institution (Universidad de Las Palmas de Gran Canaria, Canary Islands, Spain).

### Reagents and Analytes of Interest

A total of 37 chemical pollutants were determined all cat samples. The 11 OPFRs were also determined in human serum samples. We have considered also sums of groups of contaminants as follows: Sum of marker PCBs, sum of those congeners considered as markers of environmental contamination for PCBs (M-PCBs, IUPAC congeners #28, 52, 101, 138, 153, and 180); sum of dioxin-like PCBs (DL-PCBs), sum of the 12 dioxin like PCBs measured (IUPAC congeners #77, 81, 105, 114, 118, 123, 126, 156, 157, 167, 169, and 189); sum of BDEs, sum of congeners #28, 47, 85, 99, 100, 153, 154, and 183; sum of OPFRs, sum of 2-ethylhexyldiphenyl phosphate, tri(2-ethylhexyl) phosphate, tributylphosphate (TBP), triethylphosphate, tiisobutylphosphate, triphenylphosphate (TPhP), tris((2-chloro-1-chloromethyl)ethyl)phosphate, tris (2-butoxyethyl)phosphate (TBOEP), TCEP, tris (2-chloroisopropyl)phosphate, and tricresyl phosphate.

We prepared stock solutions of each compound in cyclohexane (1 mg/mL), which were stored at −20°C. Diluted solutions from 0.03 to 20 ng/mL were used for calibration curves (10 points) in cyclohexane containing 1% olive oil as analyte protectant (especially for organophosphorous compounds), as described previously (23, 24). PCB 202 and diazinon-d10 were employed as internal standards. All the standards were neat compounds and were acquired from Dr. Ehrenstorfer Reference Materials (Augsburg, Germany). All the organic solvents were of mass spectrometry grade (Merck, Darmstadt, Germany), and the ultrapure water was produced in the laboratory using a Milli-Q Gradient A10 apparatus (Millipore, Molshein, France). The rest of the reagents employed in this work were acquired from Sigma-Aldrich (St. Louis, USA).

### Extraction and Clean-up Procedure

One-milliliter aliquots of plasma samples were subjected to solid-phase extraction as previously reported for animal serum samples (25, 26). Briefly, samples were mixed with 1 mL of water/n-propanol (prepared in a proportion 85/15, v/v) and applied to 200 mg (3 mL) Chromabond^®^ C18ec columns (Macherey-Nagel, Germany). These solid-phase extraction columns were mounted in a manifold (Waters Corporation, USA), which was operated under vacuum. Fist, the columns were cleaned and conditioned using 2 mL methanol and 2 mL of water/n-propanol at a constant flow rate of 1.5 mL/min. After the application of serum samples, the columns were washed two times with water/n-propanol (500 µL), and the columns were allow to dry leaving them during 30 min under vacuum. The elution of the compounds of interest was performed with two volumes of cyclohexane (250 µL each). Finally, the solvent of the extract were evaporated to dryness under a nitrogen stream. The dry residues were then re-solubilized in 200 µL of cyclohexane (27) and employed in the subsequent chromatographic analyses. Analyte recoveries were good, as all were in the range 89–107%.

### Procedure of Chemical Analysis

We employed a GC System 7890B equipped with a 7693 Autosampler (Agilent Technologies, Palo Alto, CA, USA) for gas chromatographic separations. Two fused silica ultra-inert capillary columns Agilent J&WHP-5MS (Crosslinked 5% phenylmethylpolysiloxane, Agilent Technologies) each with a length of 15 m, 0.25 mm i.d., and a film thickness of 0.25 µm were connected in series and used as the stationary phase. Both columns were connected by a Purged Ultimate Union (PUU; Agilent Technologies). Helium (99.999%) at a constant flow rate of 1.0 mL/min for column 1 was used as the carrier gas. We employed the back-flushing technique during the chromatographic separations. In this technique the low volatility analytes and matrix components that are retained at the head of the column because of their low mobility under the chromatographic conditions used are eliminated through a purge valve. This is done by reversing the helium flow in the first chromatographic column once the last analyte of interest has been eluted from it. The oven temperature program was programmed as follows: (a) 60°C held for 1 min; (b) increase to 170°C at a rate of 40°C/min; (c) increase to 310°C at a rate of 10°C/min to 310°C; (d) 3 min hold time; and (e) cool down to 60°C. Each chromatographic analysis lasted 20.75 min. Injector and transfer line were set at 280°C. Standards and samples were injected (1 µl) in the splitless mode using a 4-mm ultrainert liner with glass wool (Agilent Technologies).

The detection of the analytes was performed using a Triple Quad 7010 mass spectrometer (Agilent Technologies, Palo Alto, CA, USA). We employed chlorpyrifos-methyl (Rt = 9.143 min) as the time reference to adjust the retention times of the analytes in this method. For this we employed the Retention Time Locking feature of the Agilent equipment used. All the analytical details of this method have been previously published (23). Nitrogen (99.99%) was used as the collision gas. Collision gas flow was set at 1.5 mL/min. The QqQ mass spectrometer was operated under the previously described conditions (28). The quantification was done using ten-point calibration curves. These calibration curves were constructed using a least-squares linear regression from the injection of standard solutions (1% olive oil), ranging from 0.03 to 20 ng/mL. The limits of quantification varied among compounds and ranged from 0.03 to 0.15 ng/mL (23, 28). The results of this study have been expressed in nanogram contaminant per gram of lipid weight (ng/g lw) for the normalization of the results.

### Quality Assurance and Quality Control (QA/QC)

Each sample was analyzed in duplicate and the means were used for the calculations. Each 16 vials (8 samples) we included 3 controls: (a) a reagent blank consisting of cyclohexane with 1% olive oil; (b) a vial consisting of cyclohexane containing 1% olive oil, but also containing a concentration of 2 ng/mL of each of the analytes of interest; and (c) a procedural blank consisting of a sample of lyophilized human serum (Medidrug Basis Line, Medichem, Germany), which was reconstituted and fortified at a concentration of 10 ng/mL of each analyte under study and extracted using same procedure described above for the samples. The results of the analyses were considered acceptable since the concentration of the analytes of the procedural blank were in all cases within 15% of the deviation from the theoretical value.

### Statistical Analysis

For the management of the database and statistical analyses, we employed PASW Statistics v 19.0 (SPSS Inc., Chicago, IL, USA). We checked the normality of the data the Kolmogorov–Smirnov test. As the distributions of the pollutants lacked normality and homoscedasticity, we used non-parametric tests (the Mann–Whitney and Kruskal–Wallis tests) in the subsequent analyses. The relationships between the categorical variables were examined by means of the chi-square test. The results were reported as mean ± SD and median. Probability levels of less than 0.05 (two-tailed) were considered statistically significant.

## Results

### Serum Levels of PCBs in Pet Cats and Humans

A total of 18 PCBs—6 M-PCBs and 12 DL-PCBs—were measured in the serum of 22 pet cats and 22 humans included in the study. A total of six compounds were detected in the series of cats (33.3%), although four of them were detected in ≤10% of pet cats (Table 1). In the case of humans, only five congeners were detected, and three were detected in more than 70% of samples. In the case of cats, only PCB-138 (detected in 50% of the series) and PCB-180 (detected in 18.2% of the series)—both considered as M-PCBs—were measured in a significant number of subjects. In fact, the highest concentrations were observed for these two congeners (138 and 153), which accounted for almost half of the measured total PCBs concentration, followed by PCBs 101 and 180, and all of them are not dioxin-like PCBs (M-PCBs). Median of sum M-PCBs was 0.35 ng/g fat, similar to the median of sum Total-PCBs. Thus, the contribution of DL-PCBs to the body burden was scarce in cats (Table 1). The concentrations detected in human serum were significantly higher for PCB-138, PCB-153, PCB-180, and for the sum of M-PCBs and total PCBs. The median value of the total sum of PCBs was around 50 times higher in human serum than in cat serum.

**Table 1:** **Concentrations of polychlorinated biphenyls (ng/g fat) in the whole series of cats (*n* = 22) and humans (*n* = 20)**.

Congener	Pet cats					Humans					*P*
	*n*	(%)	Mean	SD	Median	*n*	(%)	Mean	SD	Median	
PCB-28	0	0	–	–	–	0	0	–	–	–	–
PCB-52	0	0	–	–	–	0	0	–	–	–	–
PCB-77	0	0	–	–	–	0	0	–	–	–	–
PCB-81	0	0	–	–	–	0	0	–	–	–	–
PCB-101	2	9.1	0.96	3.52	0.00	0	0	–	–	–	–
PCB-105	0	0	–	–	–	0	0	–	–	–	–
PCB-114	0	0	–	–	–	0	0	–	–	–	–
PCB-118	1	4.5	0.16	0.75	0.00	1	5	0.04	0.12	0.00	n.s.
PCB-123	0	0	–	–	–	0	0	–	–	–	–
PCB-126	1	4.5	0.44	2.07	0.00	1	5	0.03	0.09	0.00	n.s.
PCB-138	11	50.0	8.65	33.27	0.35	18	90	4.82	4.56	3.45	0.0224*
PCB-153	2	9.1	8.95	38.27	0.00	14	70	7.66	7.58	6.05	0.0013**
PCB-156	0	0	–	–	–	0	0	–	–	–	–
PCB-157	0	0	–	–	–	0	0	–	–	–	–
PCB-167	1	4.5	0.24	1.11	0.00	0	0	–	–	–	–
PCB-169	0	0	–	–	–	0	0	–	–	–	–
PCB-180	4	18.2	0.67	2.19	0.00	16	80	9.44	9.21	6.45	<0.0001****
PCB-189	0	0	–	–	–	0	0	–	–	–	–
∑M-PCBs	11	50.0	20.08	78.31	0.35	18	90	21.92	21.14	15.95	<0.0001****
∑DL-PCBs	2	9.1	0.84	3.23	0.00	2	10	0.23	0.71	0.00	n.s.
∑Tot-PCBs	11	50.0	20.08	78.31	0.35	18	90	22.15	21.62	15.95	<0.0001****

*P < 0.05; **P < 0.01; ****P < 0.001.

*PCBs, polychlorinated byphenils*.

*∑M-PCBs: sum of marker PCBs (IUPAC congeners #28, 52, 101, 138, 153, and 180)*.

*∑DL-PCBs: sum of dioxin-like PCBs (IUPAC congeners #77, 81, 105, 114, 118, 123, 126, 156, 157, 167, 169, and 189)*.

*∑Tot-PCBs: sum of all 18 PCBs cited above*.

### Serum Levels of BDEs in Pet Cats and Humans

A total of 8 BDEs—tri-BDEs (congener #28), tetra-BDEs (congener #47), penta-BDEs (congeners #85, 99, and 100), hexa-BDEs (congeners #153 and 154), and hepta-BDEs (congener #183)—were measured in the serum of 22 pet cats and 20 humans included in the study. In cats, a total of five compounds were detected (62.5%), although four of them were detected in ≤10% of pet cats (Table 2). Only two of the compounds detected in cat serum were also detected in human serum samples. BDE-153 was detected in 10% of human samples and was not detected in cats. Only BDE-47 was measured in a significant number of subjects (*n* = 19, 86.4% in cats; *n* = 10, 50% in humans). Median values of BDE-47 were 3.75 ng/g fat in cat serum, and 0.90 ng/g fat in human serum, and this congener was the main contributor to the total body burden of BDEs in both species (4.45 and 2.2 ng/g fat in cats and humans, respectively, Table 2). The median value of the total sum of BDEs was around half in human serum than in cat serum.

**Table 2:** **Concentrations of brominated diphenyl ethers (ng/g fat) in the whole series of cats (*n* = 22) and humans (*n* = 20)**.

Congener	Pet cats					Humans					*P*
	*n*	(%)	Mean	SD	Median	*n*	(%)	Mean	SD	Median	
BDE-28	0	0	–	–	–	0	0	–	–	–	–
BDE-47	19	86.4	4.88	4.36	3.75	10	50	1.34	1.42	0.9	0.0002***
BDE-85	2	9.1	0.08	0.25	0.00	0	0	–	–	–	–
BDE-99	1	4.5	0.09	0.41	0.00	0	0	–	–	–	–
BDE-100	0	0	–	–	–	0	0	–	–	–	–
BDE-153	0	0	–	–	–	2	10	0.28	0.86	0.00	–
BDE-154	1	4.5	0.18	0.83	0.00	0	0	–	–	–	–
BDE-183	2	9.1	0.26	1.00	0.00	0	0	–	–	–	–
∑BDEs	23	95.5	5.48	4.35	4.45	13	65	1.62	1.41	2.2	<0.0001****

***P < 0.005; ****P < 0.001.

*BDEs, brominated diphenyl ethers*.

### Serum Levels of OPFRs in Pet Cats and Humans

To our knowledge, this is the first time describing serum levels of OPFRs in pet cats. A total of 11 compounds were measured in the serum of 22 pet cats and 20 humans included in the study. A total of six compounds were detected in 100% of the samples, and other one was detected in 82% of cats and 90% of humans (TBOEP). Only one compound, tris ((2-chloro-1-chloromethyl)ethyl) phosphate, was not detected in any cat (Table 3). However, it is striking the case of tricresyl phosphate, which was detected in 77.3% of cats and in none of the human samples. 2-Ethylhexyldiphenyl phosphate and tris (2-chloroisopropyl) phosphate were the pollutants detected at the highest concentrations in both species. On the other hand, triethylphosphate, detected in 50% of pet cats, was the pollutant with lowest concentration (median concentrations of 0.60 ng/g fat in cats and 0.00 ng/g fat in humans). Sum OPFR in cats was 909.25 ng/g fat and 877.72 ng/g fat in humans. These results of cats and humans were not significantly different. The median values of the sum OPFRs reported here is at least 200 times higher than serum levels of BDEs and almost 2,500 times higher than median values of PCBs.

**Table 3:** **Concentrations of organophosphorous flame retardants (ng/g fat) in the whole series of cats (*n* = 22) and humans (*n* = 20)**.

Congener	Pet cats					Humans					*P*
	*n*	(%)	Mean	SD	Median	*n*	(%)	Mean	SD	Median	
2-Ethylhexyldiphenyl phosphate	22	100	617.64	273.30	535.20	20	100	177.6	567.3	425.8	0.0008***
Tri(2-ethylhexyl) phosphate	6	27.3	0.53	1.34	0.00	10	50	1.15	1.49	0.4	n.s.
Tributylphosphate	22	100	93.13	62.46	72.80	20	100	58.26	19.06	64.80	n.s.
Triethylphosphate	11	50.0	2.96	6.93	0.60	6	30	1.54	3.22	0.00	n.s.
Triisobutylphosphate	22	100	77.05	72.92	48.50	20	100	48.66	21.32	47.70	n.s.
Triphenylphosphate	22	100	29.58	15.39	24.65	20	100	18.52	6.83	22.67	n.s.
Tris ((2-chloro-1-chloromethyl)ethyl)phosphate	0	0	–	–	–	0	0	–	–	–	–
Tris (2-butoxyethyl)phosphate	18	81.8	53.08	42.00	44.90	18	90	106.3	149.8	56.41	n.s.
Tris (2-chloroethyl)phosphate	22	100	12.10	17.43	7.15	20	100	3.12	1.27	3.69	0.0003***
Tris (2-chloroisopropyl)phosphate	22	100	150.40	111.97	114.45	20	100	83.65	28.94	93.97	n.s.
Tricresyl phosphate	17	77.3	13.41	12.91	10.40	0	0	–	–	–	–
∑OPFRs	22	100	1049.88	558.93	909.25	22	100	712.12	304.8	877.72	n.s.

***P < 0.005.

*OPFRs, organophosphorous flame retardants*.

**Figure 1:**
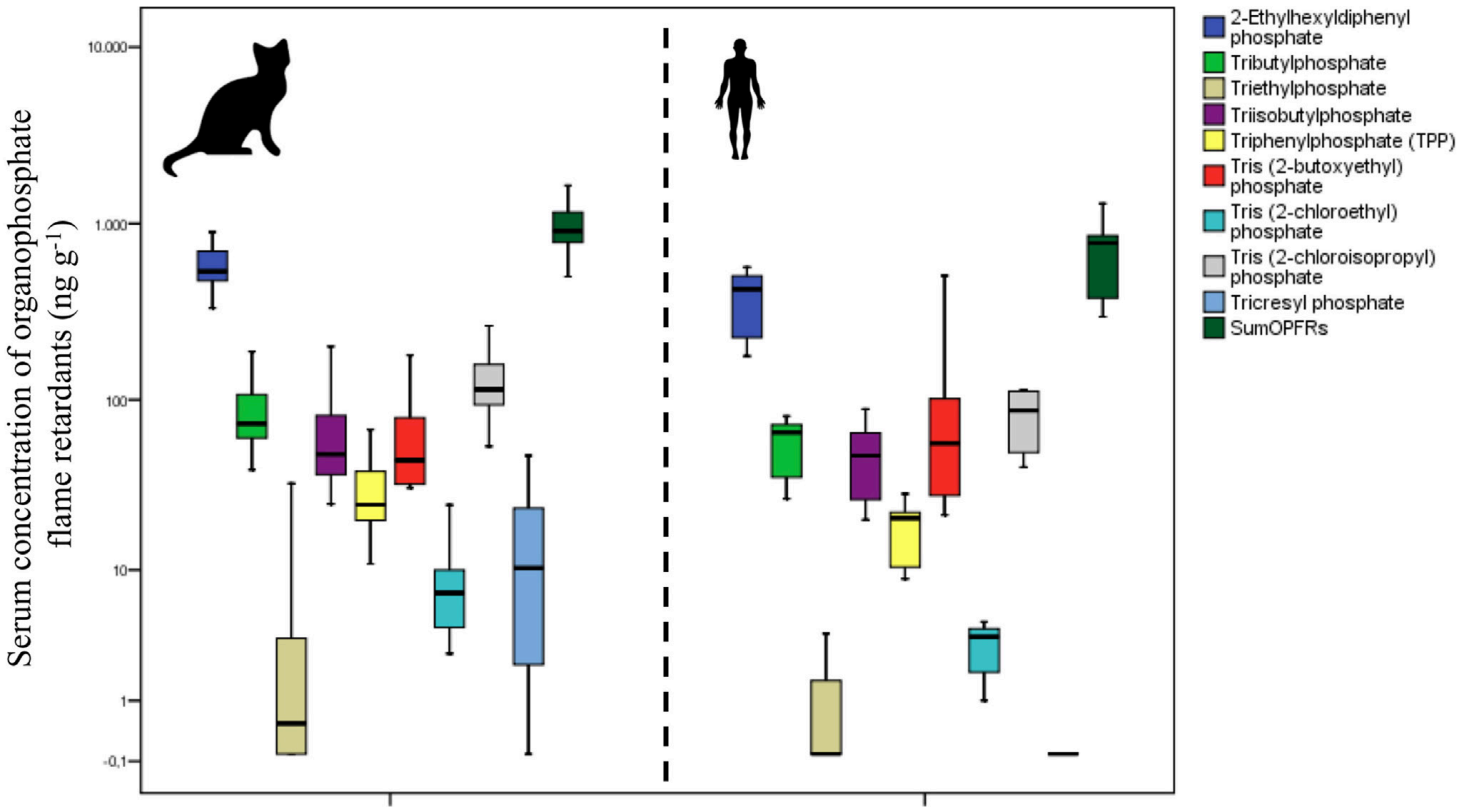
**Box plot showing the serum levels of the OPFRs detected in ≥50% of the samples, among cats (*n* = 22) and humans (*n* = 20)**. The lines connect the medians, the boxes cover the 25th to 75th percentiles, and the minimal and maximal values are shown by the ends of the bars.

**Figure 2:**
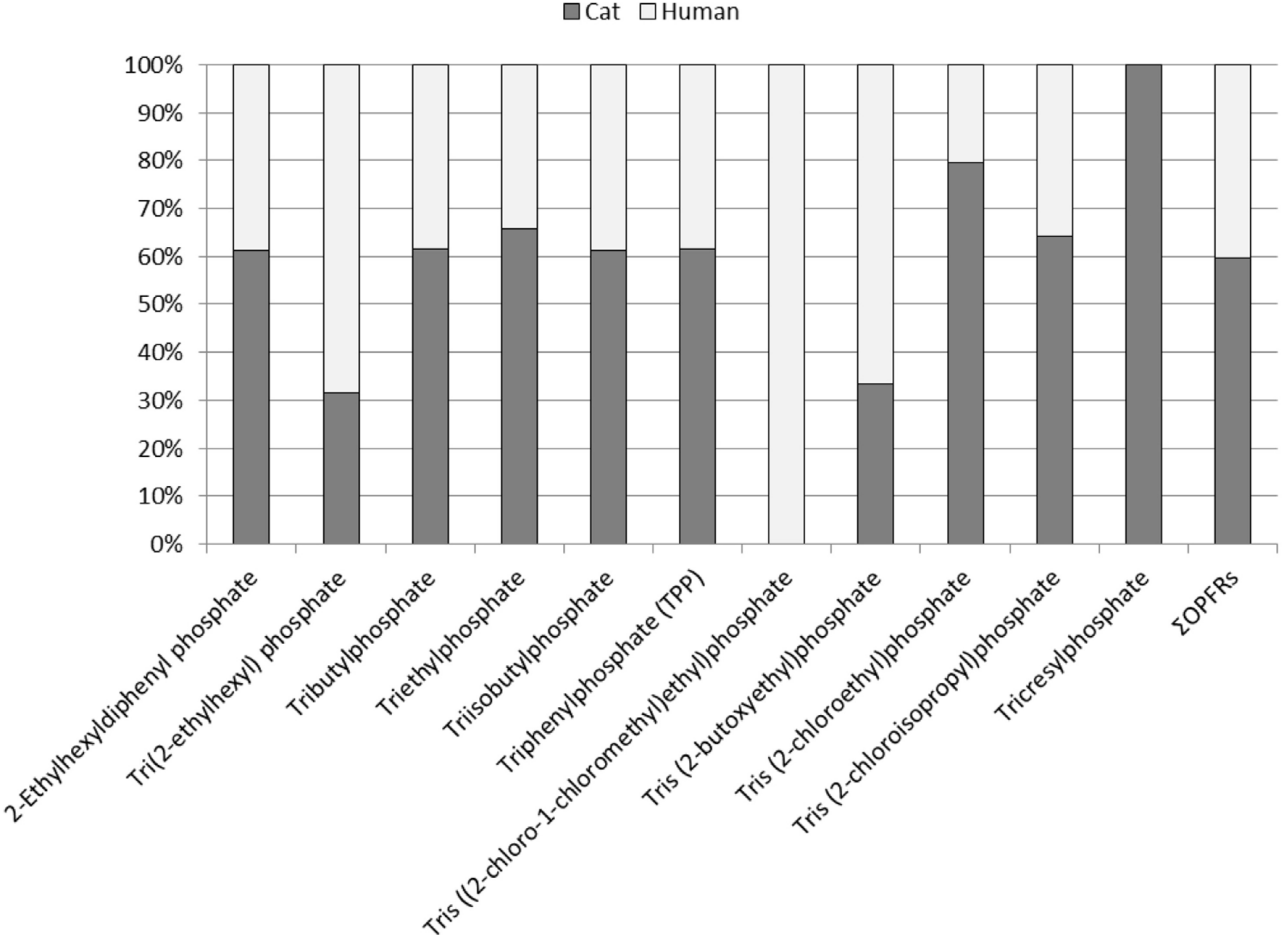
**Stacked bar graph showing the proportionality of the amount of each OPFR between cat and human serum (expressed as percentage)**.

When we compared the concentrations of OPFRs found in the serum of cats with those found in human serum we found a similar profile of distribution (Figure 1) between both species. The levels in humans were slightly lower in human serum than in cat serum, and for two of these compounds the differences were statistically significant (Table 3). However, as shown in Figure 2, the grouped bar graph of the averaged concentrations found in both species show that the proportions were similar for the majority of these compounds, except for tricresyl phosphate, which was found only in cat samples.

## Discussion

Serum levels of different types of flame retardants—including PCBs, BDEs, and OPFRs—were determined in a cohort of pet cats aimed to describe the exposure of such animals to these compounds, especially regarding to OPFRs, which have been less studied in this species. The same compounds were determined in the serum of 20 pet cat owners (although they were not the owners of the cats included in this study, due to logistical limitations), with the aim of comparing the concentrations found, and to disclosing whether pet cats may serve as sentinels of human exposure to these pollutants.

Regarding the group of legacy flame retardants—PCBs—only PCB 138 and PCB 180 were detected in a significant number of subjects and at very low concentrations. The profile of contamination by these legacy flame retardants is very similar between cats and humans, but the concentrations found in the latter are several times higher. Besides, PCB congeners were also more frequently detected in human beings than in pet cats.

The profile of PCB contamination we found in this study is very similar to those reported in other previous studies in cats. However, the median values reported here were much lower than those in previous studies. Thus, in a cohort of 20 cats from Pakistan, PCB congeners (101, 118, 138, and 153) were detected in ≥70% of the series (29); and, while in that study a median value for ∑PCBs of 36 ng/g lipid weight was reported, in this study we have found a median value which is 100 times lower (0.35 ng/g lipid weight). It is probable that in Pakistan a more recent and continued exposure to obsolete electric and electronic equipment (EEE) and the waste they generate (WEEE) have existed. Until mid 1980s, EEE used to contain high amounts of PCBs, as they were employed as flame retardants in those devices. It has been reported that in certain areas (especially urban areas) of developing countries, exposure to PCBs is high due to the exposure to WEEE (11). Also in previous studies in cats from France (13) or USA (19), the cats also exhibited higher serum levels of PCBs, and also a higher variety of congeners detected. However, it has been reported that a geographical gradient in the level of PCB contamination exists, and the pattern varies greatly from region to region (30). Thus, the apparent discrepancy in the level of PCB contamination in cats from the Canary Islands with those from other parts of the world has a parallelism with the values observed in humans from this archipelago, in which also lower values of PCBs than those in other populations have been reported (12). It should be noted that the economy of the Canary Islands is based fundamentally on a few economic sectors: tourism and, to a much lesser extent, farming and fishing, and that other economic sectors, such as traditional polluting industries, have a limited presence in the Islands (31), contributing to explain the low levels of PCBs detected in human and pet cats. In any case, serum levels of ∑PCBs were the lowest among all the FRs included in this study, which is logical because, although these are very persistent chemical, they were banned for 40 years. This result is consistent with those published by other authors worldwide (12, 13, 29). Although the comparison between cats and humans indicate that the profile of contamination in very similar between both species, pet cats exhibited much lower concentrations of those. Thus, pet cats do not seem to be good sentinels of the human exposure to this group of pollutants, as previously reported also for pet dogs (28, 32, 33).

With respect to the second most abundant group of flame retardants in cat serum—the BDEs—it is important to note that, similarly to what occur with PCBs, most of them have been banned, although more recently than PCBs. Due to this ban, different authors have reported a decreasing trend in the environmental and serum levels along the last decade. Currently, the only compound of the BDE family that is still approved for use is BDE-209 (www.pops.int), and it has to be taken into account that unfortunately this chemical was not included in this study, because it can not be determined in our laboratory due to technical limitations. In any case, and even without having included the BDE 209, our results are very similar to those reported in cats from Pakistan (median ∑BDEs = 6.1 ng/g fat) (29), USA—Georgia, Massachusetts, and North Carolina—(median ∑BDEs = 5.9 ng/g fat) (17), but lower than those reported in Sweden (median ∑BDEs = 24.1 ng/g fat) (34), and than in those cats from California, in which the authors reported extraordinarily high levels of BDEs (median ∑BDEs = 2904 ng/g fat), mainly due to the high levels of BDE-99 detected in those animals (19). While diet, mainly through fish intake, is considered the main route of exposure for PCBs (23, 35–37), the ingestion of dust from the environment is considered the most important route of exposure for BDEs in cats (38). This may explain why, although the profile of contamination between humans and cats are very similar, the concentrations of congeners are slightly (and significantly) higher in cats than in humans. Thus, due to their grooming habits, cats have been classically used to evaluate the level of human exposure to BDEs through the indoor dust (39). In this sense, a positive correlation between serum cat levels and dust has been consistently reported in the literature (34), although the serum levels tend to be several times higher in cats than in humans. Compared to humans, ∑BDE levels in cats are often 20- to 100-fold greater than median levels in US adults (17, 38), but the correlation of levels and the pattern of contamination (congener distribution and proportions) between both species has been reported to be very good. In our study this pattern is maintained, and the concentrations found are much more similar between both species than those reported in other studies (median value of sum BDEs is only double in cats than in humans of this study). Hence, pet cats have been proposed as sentinels to better assess human exposure and adverse health outcomes related to low level but chronic BDE exposure (17). Our study is consistent with previous ones and seems to reinforce the hypothesis that pet cats are good sentinels for human exposure to this group of flame retardants.

Finally, with regard to the group of flame retardants most abundant in cat and human serum, the OPFRs, it should be noted that to date there are very few studies that have determined them in blood or serum of higher vertebrates, and as far as we know this is the first report in cats and, as well as one of the few available in humans. Despite this limitation, our results agree with the scarce data available in humans, which have reported a high frequency of detection (70–100%) of TBOEP, TPhP, TCEP, and TBP (40). High amounts of OPFRs have been detected in dust from houses (41), schools (42), and other everyday places (43). According to our results, when comparing cats to humans, the profile of exposure was comparable among both species (Figures 1 and 2). Although some compounds, similarly to what occurs with BDEs, were found at higher concentrations in serum of pet cats than in human serum, in general terms both patterns of exposure were virtually overlapped (Figure 1), suggesting that cats may play a potential role of cats as sentinels of the human exposure to OPFRs. Although the concentrations of some of these compounds were significantly different between cats and humans, the differences in concentration were relatively small, and when we summed the concentrations of all of the OPFRs the significance disappeared. However, it is worth to mention the case of TCP, because this compound was detected at a very high concentration in cats, but it was completely absent in humans (Figure 2). It is not easy to find an explanation to this discrepancy, because of the lack of supporting bibliography. It is worth to note, however, that the reports on environmental levels of OPFRs are extremely variable, not only regarding the concentrations, but also in the compounds mixture. In the case of TCP, it has been reported that this compound is frequently associated with urban areas, where a high disposal of WEEE exists (44). However, due to the wide range of current applications of OFPRs and the wide variety of mixtures employed, reports for TCP and other chemicals may vary from undetected to account for more than 50% of the ∑OPFR (45, 46). This could explain the disparities observed among studies (especially regarding to amount of chemicals detected, but also the composition of the mixtures). Taking this into account, a plausible hypothesis to explain the discrepancy between cats and cat owners of this study is the fact that, due to logistical reasons, we could not recruit serum from the cats and their owners. In this case, the humans recruited for this study were cat owners but not the owners of these cats, and therefore the samples of both species may not be directly compared. However, despite this limitation, we think that the findings are of interest due to the global contamination of OPFRs and the daily exposure to these compounds. Besides, our results allow to suggest that cats are good sentinels, not only of human exposure to BDEs, but also to OPFRs. Moreover, pet cat and human exposures to OPFRs are of concern since they may pose a risk for health [i.e., TCEP is classified as carcinogen (category 3) (47)].

## Conclusion

Serum levels of different flame retardants were measured in serum of pet cats and cat owners. Our results showed that PCBs, banned 40 years ago, showed the lowest levels of exposure in both species, although the levels in humans were almost 50 times higher. The frequencies of detection and levels were followed by BDEs, which were banned much more recently. In the case of the compounds within this group the profile of contamination between humans and cats were very similar, and also the concentrations were closer, although higher in cats. Finally, OPFRs were frequently detected, and at the highest concentrations in our series of cats and humans and both, the pattern of detection and concentrations were similar between both species. Our study indicates that, unlike what occurs for PCBS, pet cats are good sentinel of human exposure to BDEs and OPFRs. It seems that further research is needed to elucidating the biotransformation routes of cats and humans, as well as studies aimed to disclose, which is toxicological relevance of such a high exposure to potentially toxic chemicals.

## Ethics Statement

The study was approved by the ethics committee of the University of Las Palmas de Gran Canaria. Informed consent was obtained from human participants. Informed consent was obtained from animal owners.

## Author Contributions

LH-H: laboratory analysis of PCBs, first draft preparation, and manuscript approval. EC: sample obtention, clinical examination of cats, and manuscript revision and approval. MC: laboratory analysis of BDEs, first draft preparation, and manuscript revision and approval. JM-A: sample obtention, clinical examination of cats, and manuscript revision and approval. LB: collaboration in manuscript preparation, design of figures, and manuscript revision and approval. VBM: sample preparation and manuscript revision and approval. YFC: sample preparation and manuscript revision and approval. SFC: sample preparation and manuscript revision and approval. MZ: laboratory analysis of OPFRs and manuscript revision and approval. OL: study design and supervision and manuscript preparation and approval.

## Conflict of Interest Statement

The authors declare that the research was conducted in the absence of any commercial or financial relationships that could be construed as a potential conflict of interest.
